# 3D Hyperspectral Data Analysis with Spatially Aware Deep Learning for Diagnostic Applications

**DOI:** 10.1021/acs.analchem.4c05549

**Published:** 2025-04-03

**Authors:** Ruihao Luo, Shuxia Guo, Julian Hniopek, Thomas Bocklitz

**Affiliations:** † Institute of Physical Chemistry (IPC) and Abbe School of Photonics (ASP), Friedrich-Schiller-Universität Jena, Helmholtzweg 4, 07743 Jena, Germany; ‡ Leibniz Institute of Photonic Technology (IPHT), Albert-Einstein-Straße 9, 07745 Jena, Germany

## Abstract

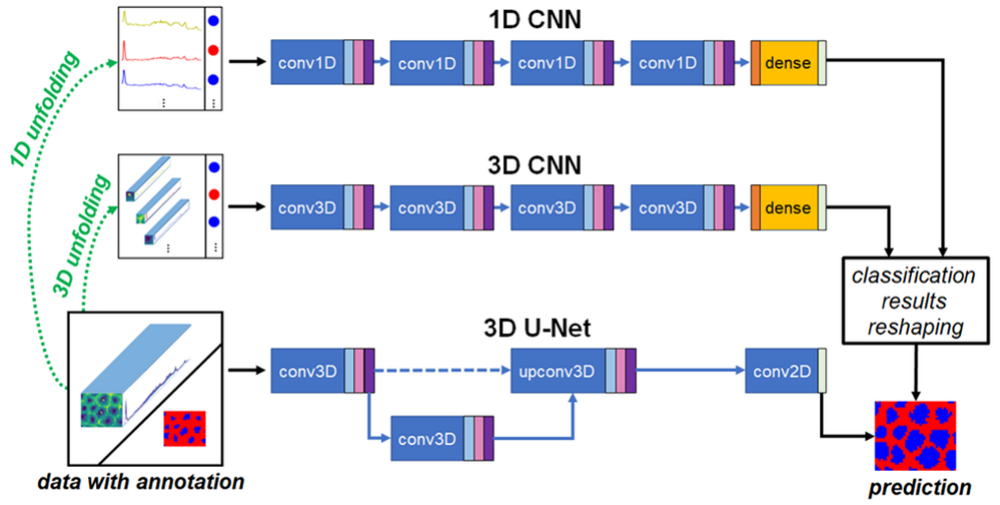

Nowadays, with the rise of artificial intelligence (AI), deep learning algorithms play an increasingly important role in various traditional fields of research. Recently, these algorithms have already spread into data analysis for Raman spectroscopy. However, most current methods only use 1-dimensional (1D) spectral data classification, instead of considering any neighboring information in space. Despite some successes, this type of methods wastes the 3-dimensional (3D) structure of Raman hyperspectral scans. Therefore, to investigate the feasibility of preserving the spatial information on Raman spectroscopy for data analysis, spatially aware deep learning algorithms were applied into a colorectal tissue data set with 3D Raman hyperspectral scans. This data set contains Raman spectra from normal, hyperplasia, adenoma, carcinoma tissues as well as artifacts. First, a modified version of 3D U-Net was utilized for segmentation; second, another convolutional neural network (CNN) using 3D Raman patches was utilized for pixel-wise classification. Both methods were compared with the conventional 1D CNN method, which worked as baseline. Based on the results of both epithelial tissue detection and colorectal cancer detection, it is shown that using spatially neighboring information on 3D Raman scans can increase the performance of deep learning models, although it might also increase the complexity of network training. Apart from the colorectal tissue data set, experiments were also conducted on a cholangiocarcinoma data set for generalizability verification. The findings in this study can also be potentially applied into future tasks regarding spectroscopic data analysis, especially for improving model performance in a spatially aware way.

## Introduction

Vibrational Raman scattering is the inelastic scattering of photons by at the vibrational states within molecules, and it was first discovered in 1928.^1^ Notably, Raman spectra can be considered as “vibrational fingerprints” of particular molecules. Therefore, these spectra can be used to distinguish corresponding biological samples when Raman spectroscopy is implemented to molecule mixtures.^2^ Nowadays, a wide range of research domains have already implemented Raman spectroscopy, for example, material identification,^3,4^ forensic analysis,^5,6^ pharmaceutical design,^7,8^ disease diagnosis,^9,10^ etc. However, due to the fact that Raman effect is selective but not sensitive, analytical models are usually necessary for interpreting the untargeted spectral data statistically.^2,11^

Models for analyzing Raman spectroscopy vary from classical machine learning methods to deep learning networks.^12^ In terms of classical machine learning, principal component analysis (PCA), k-nearest neighbors (k-NN), support vector machine (SVM), as well as random forests are very popular methods.^13^ For example, Guo et al., built a biological classifier with Raman spectra using a modified PCA;^14^ Othman et al. developed a reduced featured k-NN to classify dengue fever from salivary Raman spectra;^15^ Chen et al. combined Raman spectroscopy with SVM for primary Sjögren syndrome (pSS) diagnosis;^16^ Zhang et al. identified brain tissues with random forests and Raman spectroscopy.^17^ With the rise of artificial intelligence (AI) in recent years, Raman spectral data analysis not only employs the classical machine learning methods above, but also deep learning algorithms. Based on their purposes, these algorithms can be categorized into preprocessing, classification, spectral highlighting, etc.^12^ For example, Neo et al. developed a novel PolymerSpectraDecisionNet (PSDN) using Raman and infrared data sets to increase plastic recycling rates;^18^ Tseng et al. proposed a vision transformer (ViT) with surface-enhanced Raman scattering (SERS) for rapid bacterial identification;^19^ Wang et al. achieved precise recognition of Carbapenem-resistant Enterobacteriaceae (CRE) using an improved residual network (ResNet) and Raman spectroscopy;^20^ He et al. invented a lightweight model named RepDwNet for special biological blood Raman spectra analysis;^21^ Jensen et al. realized label-free blood typing by Raman spectroscopy with their convolutional neural network (CNN).^22^ The majority of current deep learning networks applied into Raman spectroscopy are 1-dimensional (1D), particularly 1D CNNs.^12^

In spite of their successes, only using 1D spectral data may lead to suboptimal results. Therefore, some researchers also attempted to incorporate 2-dimensional (2D) neural networks into Raman data. For example, He et al. implemented a variational autoencoder (VAE) to downscale high-dimensional Raman spectral data into 2D for accurate tumor subtype detection;^23^ Shang et al. utilized 2D CNN for polarized micro-Raman spectroscopy to detect breast cancer;^24^ Zhu et al. designed their 2D CNN based on SERS spectrograms to detect zearalenone (ZEN) in corn oil;^25^ Liu et al. used their wavelet packet transform and Gramian angular field (WPGA) algorithm to generate 2D SERS spectrograms for 2D CNN to identify bacteria.^26^ But the majority of these 2D methods rely on the mathematical transformation of 1D Raman spectra, only very few of them take spatial information into account.

The outcomes could be significantly improved by considering the neighboring information within the 3-dimensional (3D) structure of Raman hyperspectral scans. In digital image processing, it is a well known fact, spatially neighboring information is one of the most important foundations.^27^ These days, using spatial information to assist hyperspectral image (HSI) classification has already widely spread, but currently most of the related studies remain in the field of geoscience.^28−30^ For example, Gao et al. combined spatial-spectral adaptive learning with pixel-wise filtering to classify HSIs;^31^ Scheibenreif et al. proposed a method using masked image modeling and spatial-spectral transformer on hyperspectral remote sensing data;^32^ Ashraf et al. evaluated their attention 3D central difference convolutional dense network (3D-CDC Attention DenseNet) on 3 HSI data sets.^33^ Although there exist some studies using 3D deep learning for biomedical images,^34^ especially for magnetic resonance imaging (MRI) scans^35^ and computed tomography (CT) scans,^36^ unfortunately this method is still not commonly taken into consideration for Raman spectra. For this reason, to investigate the feasibility of containing spatially neighboring information on Raman spectroscopy, 3D deep learning algorithms were evaluated based on a colorectal tissue data set^37^ for both epithelial tissue detection and colorectal cancer detection in this study. First, a simplified version of 3D U-Net^38^ was utilized for whole-scan segmentation; second, a 3D CNN using 3D Raman patches was utilized for pixel-wise classification. Both methods were compared with the conventional 1D CNN method, which worked as baseline. As demonstrated by the experimental results from external K-fold cross-validation, it has been found that using spatially neighboring information on 3D Raman scans can increase the performance of deep learning models, although it might also increase the complexity of network training. Additionally, a cholangiocarcinoma data set from hyperspectral microscopy^39,40^ was also utilized in this study for generalizability verification.

## Data Set Information

### Hyperspectral Raman Data Set for Colorectal Tissue Diagnostics

To evaluate the performance of 1D and 3D deep learning networks, a colorectal tissue data set containing 3D Raman hyperspectral scans of mice was chosen for this study.^37^ In this data set, there are in total 749 scans from 156 mice, including 1,018,640 Raman spectra with 677 wavenumbers. To acquire these scans, cells were illuminated with a 785 nm diode laser, recorded in scanning mode with a step size of 5 μm and an integration time of 2 s per spectrum. And the scan size is limited to 170 × 200 μm^2^, measured with a grid size of 34 × 40 points. After acquiring these scans, a semiautomated annotation procedure was conducted based on K-means clustering together with histopathological images evaluated by a trained pathologist. With this approach, as shown in Table 1, the original data set contains Raman spectra belonging to the classes of carcinoma, adenoma, hyperplasia, normal, spectroscopic artifact, other tissue, question, and zero as well. The “question” class refers to the spectra which are without clear label information; and because some scans are smaller than 34 × 40, zeros were added to ensure all the scans are with the same size, resulting in a “zero” class. Besides, to guarantee that not just colorectal cancer types are able to be detected, but epithelial tissues can also be distinguished from other tissues and spectroscopic artifacts, a 2-stage classification was designed. In this manner, the classes of original data set were rearranged for epithelial tissue detection and colorectal cancer detection, respectively. For epithelial tissue detection, carcinoma, adenoma, hyperplasia and normal classes were defined as “epithelial tissue”, while the spectral values in the original question and zero classes were set to 0 and then defined as the new “rest class”; and for colorectal cancer detection, the spectral values in the original spectroscopic artifact, other tissue, question, and zero classes were all set to 0 and defined as ‘others’. This was done to ensure that no errors were introduced by unclear labeling (“question”) and to focus on specific tasks by excluding nonrelevant groups.

**Table 1:** Classes of the Original Dataset, Epithelial Tissue Detection, and Colorectal Cancer Detection

original data set		epithelial tissue detection		colorectal cancer detection	
class	spectra count	class	spectra count	class	spectra count
carcinoma	57,400	epithelial tissue	684,916	carcinoma	57,400
adenoma	168,415			adenoma	168,415
hyperplasia	68,552			hyperplasia	68,552
normal	390,549			normal	390,549
spectroscopic artifact	69,257	spectroscopic artifact	69,257	others	333,724
other tissue	70,452	other tissue	70,452		
question	178,883	rest class	194,015		
zero	15,132				
total	1,018,640	total	1,018,640	total	1,018,640

**Figure 1:**
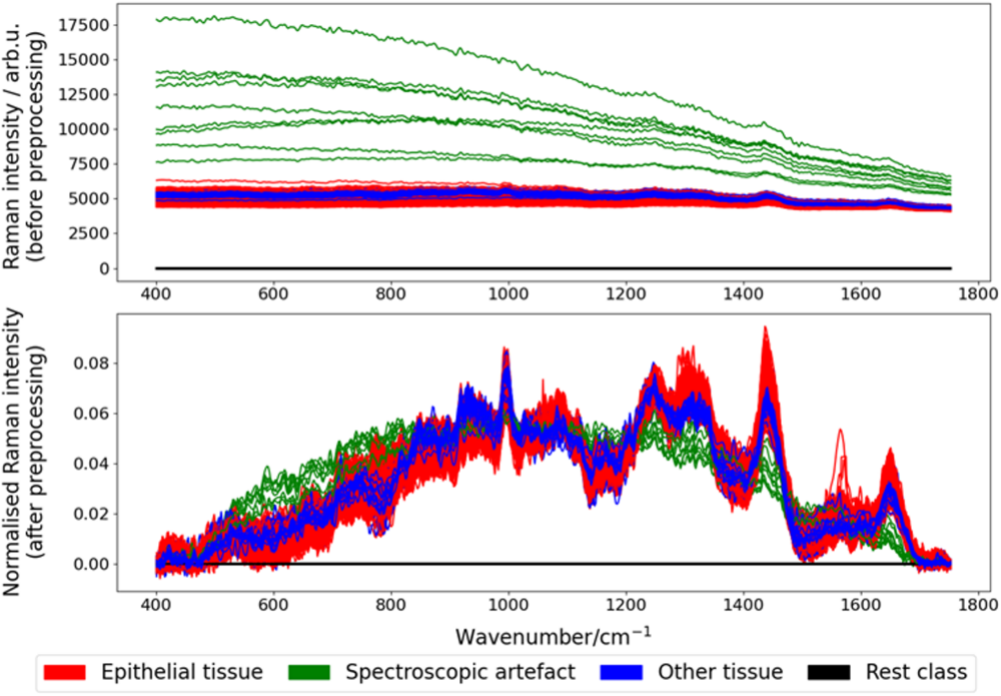
Example of spectra preprocessing. After preprocessing steps including background removal, despiking, and normalization, the quality of Raman spectra was greatly improved. These preprocessing steps made it possible for deep learning networks to obtain better results in the later classification and segmentation tasks.

Before feeding these Raman spectra into deep learning networks, they were preprocessed via RAMANMETRIX.^41^ The preprocessing contains steps of background removal, despiking, and normalization. In terms of background removal, the statistics-sensitive nonlinear iterative peak-clipping (SNIP) algorithm^42^ was employed, which assumes a smooth and polynomial-like fluorescence background. Similar to the results of many other baseline correction algorithms, some Raman and autofluorescence signals might overlap due to complex fluorescence variations. Therefore, given these practical challenges, these spectra were treated as spectroscopic artifacts to ensure the classification robustness of colorectal cancer detection, which is of greater clinical significance. Figure 1 illustrates an example of spectral data before and after preprocessing. It is clearly apparent that the preprocessed spectra had much better quality, which made it possible to obtain better results in the later classification and segmentation tasks.

### Hyperspectral Data Set of Cholangiocarcinoma Tissue

Additionally, to verify the generalizability of findings in this study, an extra cholangiocarcinoma data set was also employed.^39,40^ In this data set, the images were collected by a microscopy hyperspectral imaging (MHSI) system with an objective lens of 20× magnification, and the spectra range from 550 to 1000 nm, resulting in 60 spectral bands for each scene. There are simply 2 classes (carcinoma and normal), and each hyperspectral scan is of the size 256 × 320 × 60. Because biomedical data sets contain only very limited data in practice, just 200 samples of hyperspectral scans were randomly chosen from this data set for network performance analysis.^43^

### Experimental Design

**Figure 2:**
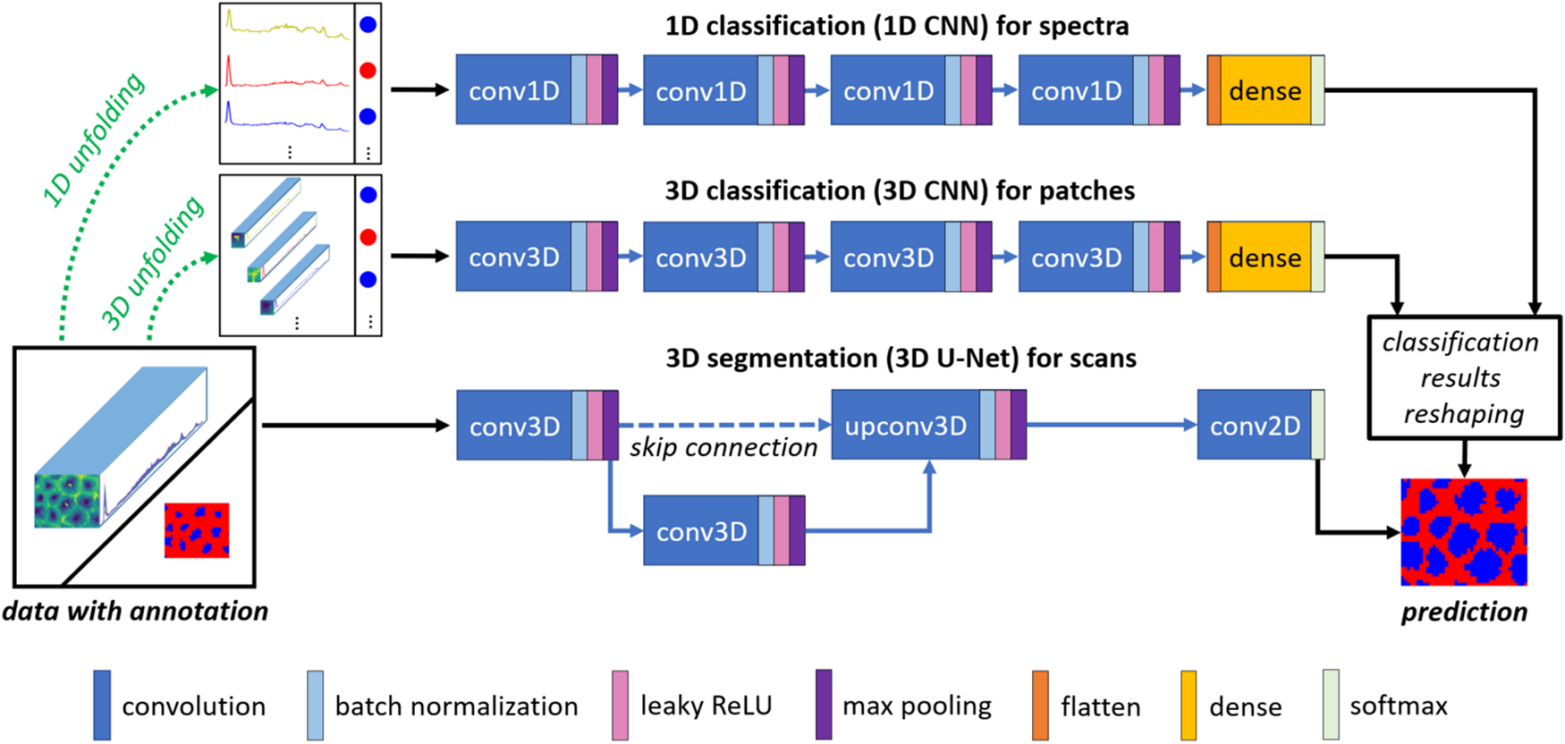
Network architectures for 3D Raman scan analysis. For 1D classification, the input 3D Raman scan is first unfolded into independent 1D spectra, and then these spectra are sent into a 1D CNN. The 1D CNN has a simple architecture with four 1D convolutional layers and one dense layer. After each convolutional layer, there are a batch normalization layer, a leaky ReLU layer and a max pooling layer as well. For 3D classification, the input Raman scan is first unfolded in a 3D way, resulting in 3D Raman patches, and each patch is annotated with the annotation of its central spectrum. Then these patches were sent to the 3D CNN, including four 3D convolutional layers and one dense layer. For both 1D CNN and 3D CNN, their pixel-wise classification results are reshaped to 2D to obtain a predicted result of segmentation. For 3D segmentation, the 3D Raman scan is directly inputted into a simplified 3D U-Net with 4 convolutional layers.

As introduced above, both 3D classification and 3D segmentation methods were compared with the conventional 1D CNN based classification method. As can be seen in Figure 2, for 1D classification, the input 3D Raman scan is first unfolded into independent 1D spectra, and then these spectra are sent into a 1D CNN. The 1D CNN adopted in this study has a simple architecture with four 1D convolutional layers and one dense layer. The four 1D convolutional layers use 128, 64, 32, and 16 kernels, respectively; and the corresponding kernel sizes are 3, 5, 7, and 9, respectively. The kernel sizes were chosen in this way because in a sequential CNN, initially a small kernel size would be preferred to capture fine details and maintain more spatial resolution, then larger kernels would be gradually employed to capture broader contextual information at deeper stages. The number of nodes in the dense layer is equivalent to the number of classes. After each convolutional layer, there are a batch normalization layer, a “leakyReLU” layer and a max pooling layer as well. Besides, its dense layer is in between a flatten layer and a softmax layer. In terms of 3D classification, the input Raman scan is first unfolded in a 3D way, resulting in 3D Raman patches with a window size of 3 × 3, and each patch is annotated according to its central spectrum. Similar to the 1D CNN, a simple 3D CNN was designed for 3D classification: it also has four convolutional layers and one dense layer, but all its convolutional layers are 3D. The four 3D convolutional layers also use 128, 64, 32, and 16 kernels, respectively; and the corresponding kernel sizes are 3 × 3 × 3, 3 × 3 × 5, 3 × 3 × 7, and 3 × 3 × 9, respectively. For both 1D and 3D classification methods, their pixel-wise classification results are reshaped to 2D to obtain a predicted result of segmentation. When it comes to 3D segmentation, a simplified 3D U-Net was implemented, which can directly process the entire 3D Raman scan and output a predicted 2D segmentation map. It has four convolutional layers as well, three of them are 3D and one convolutional layer is 2D. The 3D convolutional layers compose a downsampling path and a upsampling path connected by a skip connection using concatenation. For the experiments on the described colorectal tissue data set, all 3D convolutional layers have 128 kernels with a size of 3 × 3 × 5, and its 2D convolutional layer has a kernel number equal to the number of classes with a size of 1 × 1. Within the experiments on the cholangiocarcinoma data set, the kernel numbers of the 3D convolutional layers in the U-Net were halved to 64 due to computational complexity. Moreover, the 3 deep learning networks in this study were all compiled with Adam optimizer using a fixed learning rate of 3 × 10^–4^.^44^

**Figure 3:**
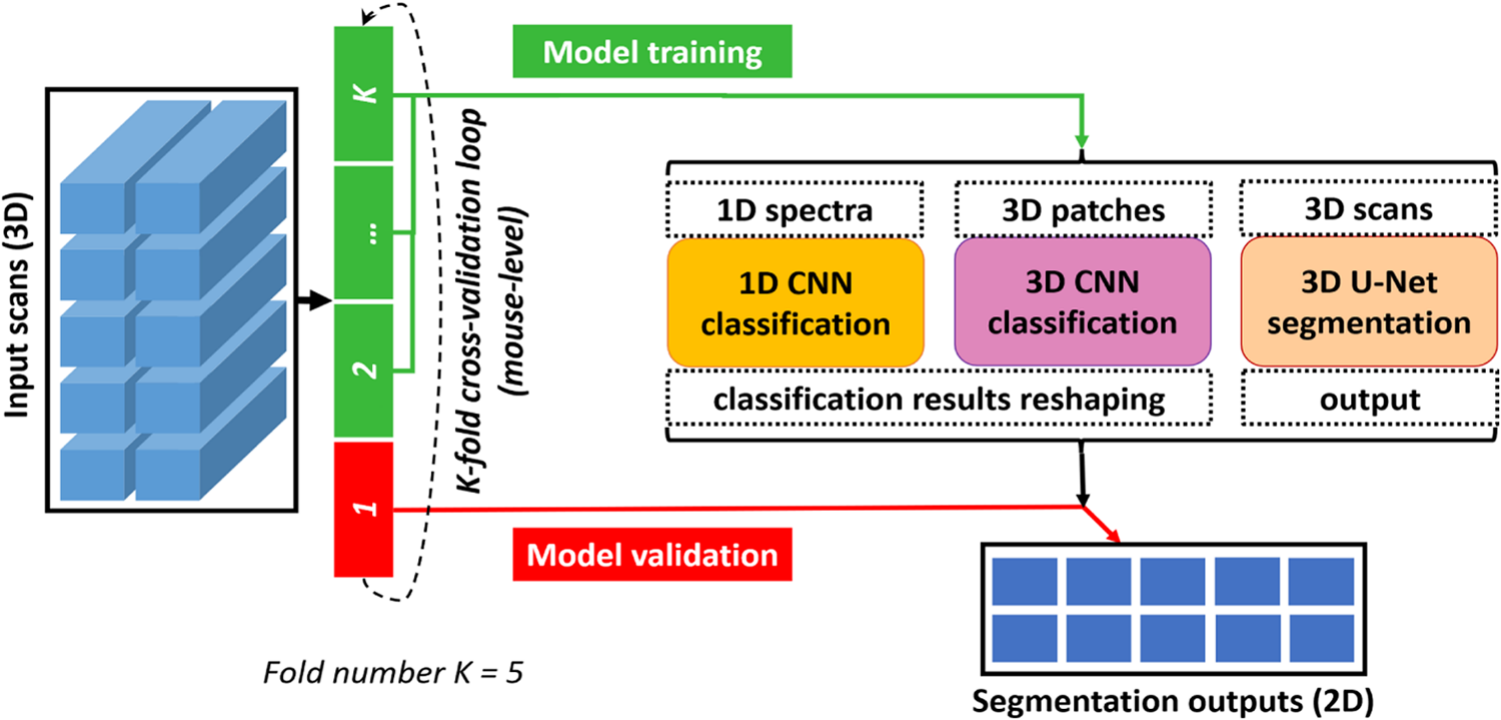
Overall dataflow of network performance analysis. With the input scans, an external K-fold cross-validation loop is first implemented based on mouse-level data set split. In each fold, the training subsets were sent to 3 deep learning networks (1D CNN, 3D CNN, and 3D U-Net), then the validation subset was used to evaluate the corresponding trained model based on the segmentation outputs. The fold number K was set as 5 in this study.

To generate enough experimental results for performance analysis, an external K-fold cross-validation loop is necessary to be put into practice in the field of biomedical research as the data sets are of limited size.^45,46^ The term external refers to the use of the cross-validation for performance estimation. Therefore, Figure 3 illustrates how this loop works for network performance analysis in this study. First, based on a mouse-level data set split, the input scans were transferred to K data subsets with an equal amount of data. Within this approach the independence of every data subset was ensured, by assigning data from one mouse only to one data subset. In each round of the cross-validation loop, one of the data subsets was chosen for validation, and the rest K-1 subsets were used for network training. Afterward, the K-1 training subsets were sent to train 3 deep learning networks; the validation subset was used to evaluate the trained model. By doing so, for each model, K validation results could be produced at the end. Following common practice, the fold number K was set to 5. It is worth noting that cross-validation was performed at the mouse level in this study: although models were built and evaluated at the spectra level, folds were assigned based on individual mouse.

## Results and Discussion

**Figure 4:**
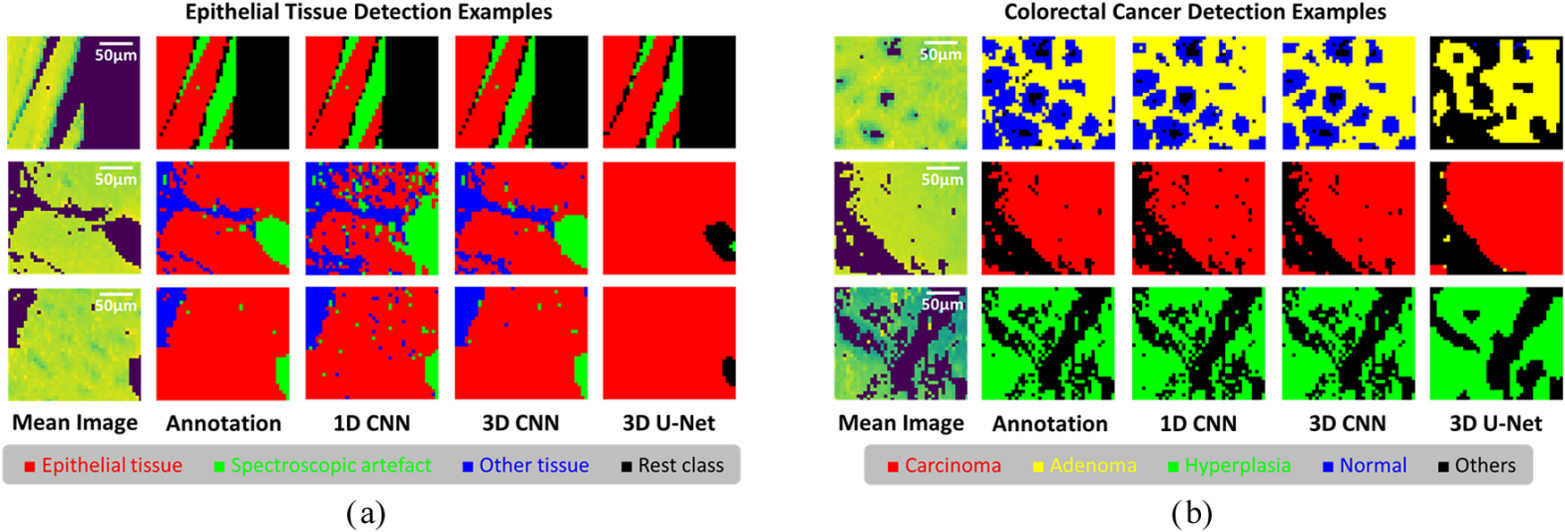
Example validation results of epithelial tissue detection (4a) and colorectal cancer detection (4b). There are 3 validation examples shown above, and each is with the mean spectral image, annotation, as well as the final segmentation outputs using 1D CCN, 3D CNN and 3D U-Net, respectively. In the epithelial tissue detection task, there are 4 classes including epithelial tissue (red), spectroscopic artifact (green), other tissue (blue), and rest class (black).

As previously mentioned, this study contains an epithelial tissue detection task as well as a carcinoma detection task using the colorectal tissue data set from mice. The experimental results of each network were obtained after 50 epochs of training using 5-fold cross-validation. With regard to epithelial tissue detection, Figure 4a shows some example results of validation. It is not difficult observe that the 1D CNN model had acceptable results in general. However, there were some single pixels wrongly classified indeed. By using spatially neighboring information on Raman hyperspectral scans, the 3D CNN model could largely reduce these artifacts and had more satisfactory results. In comparison, the 3D U-Net model performed worst, and it did not even learn enough details from the annotation, mainly due to its massive trainable parameters on a rather limited data set.

Apart from the example predictions, to assess model performance more statistically, accuracies and balanced accuracies were also calculated for each cross-validation fold based on 2D segmentation outputs using eqs 1, 2, respectively.
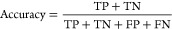
1where TP means true positives, TN means true negatives, FP means false positives, and FN means false negatives.
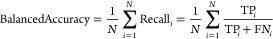
2where *N* means the number of classes, Recall*_i_* means the recall of class *i*, TP*_i_* means true positives of class *i*, and FN*_i_* means false negatives of class *i*.

**Figure 5:**
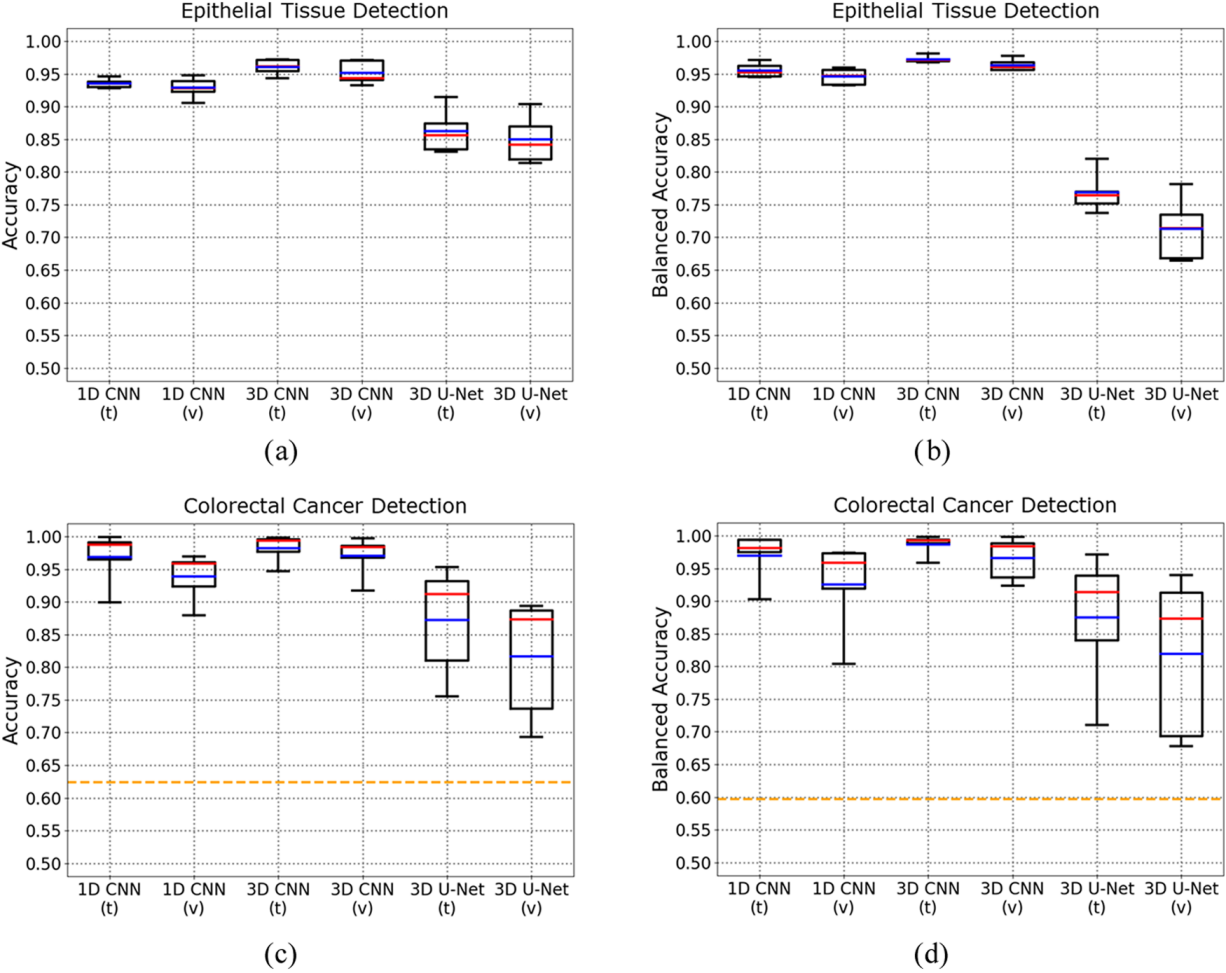
Network performance boxplots of epithelial tissue detection (5a, 5b) and colorectal cancer detection (5c, 5d). The boxplots above illustrate the training and validation performance of 1D CNN, 3D CNN, and 3D U-Net for epithelial tissue detection using accuracy (5a, 5c) and balanced accuracy (5b, 5d), respectively. In the plots, “t” refers to “training”, and “v” refers to “validation”. These values were calculated for each fold, therefore each box contains 5 values. Besides, the red lines demonstrate the average values, while the blue lines demonstrate the median values. Additionally, the orange dashed lines display the performance of Vogler et al. using SVM.^37^

Figures 5a,b demonstrate the plots of accuracies and balanced accuracies calculated based on every fold using mouse-level data splitting, which compare the network performances in a more statistical way. Notably, due to their zero spectral values, the pixels set as the “rest class” in epithelial tissue detection and the “others” in colorectal cancer detection were excluded from calculation for better comparison. In terms of accuracies, it can be seen that both 1D CNN and 3D CNN had good training performance around 0.95, while 3D U-Net had training accuracies just a bit higher than 0.85; besides, 3D CNN had the highest validation accuracies and the most stable validation performance (many above 0.95), all validation accuracies of 1D CNN were below 0.95, and most validation accuracies of 3D U-Net were just around 0.85. In terms of average values of validation balanced accuracies, those of 1D CNN, 3D CNN, and 3D U-Net are 0.946, 0.963, and 0.713, respectively. Both 1D CNN and 3D CNN also had good training performance around 0.95; but some validation results of 1D CNN were below 0.95, while 3D CNN had the best validation performance (all above 0.95) again. In contrast, the validation performance of 3D U-Net was not desirable, with results fluctuating just from about 0.68 to 0.78.

On the question of colorectal cancer detection, Figures 4b, 5c,d illustrate the results. In the same way as epithelial tissue detection, 1D CNN also had tolerable predictions in general, while 3D CNN preserved details with relatively higher quality. By contrary, due to under-fitting, 3D U-Net could not sufficiently capture the underlying patterns and relationships in the training data, causing quite poor predictive performance. Statistically, the average validation balanced accuracies of 1D CNN, 3D CNN, and 3D U-Net are 0.926, 0.966, and 0.819, respectively. Most accuracies and balanced accuracies of 1D CNN during training fluctuate between 0.90 and 1.00, while those of 3D CNN fluctuate above 0.95. And the validation balanced accuracies of 3D CNN (mostly above 0.95) were higher and more stable than those of 1D CNN (mostly below 0.95). But 3D U-Net just had training accuracies and training balanced accuracies unstably fluctuating in a much larger range, and most of its validation accuracies and validation balanced accuracies were around just 0.80. On the other hand, in most circumstances, all 3 deep learning networks outperformed the conventional method using support vector machine (SVM) adopted by Vogler et al., which was only 0.62 for accuracy and 0.60 for balanced accuracy.^37^

**Figure 6:**
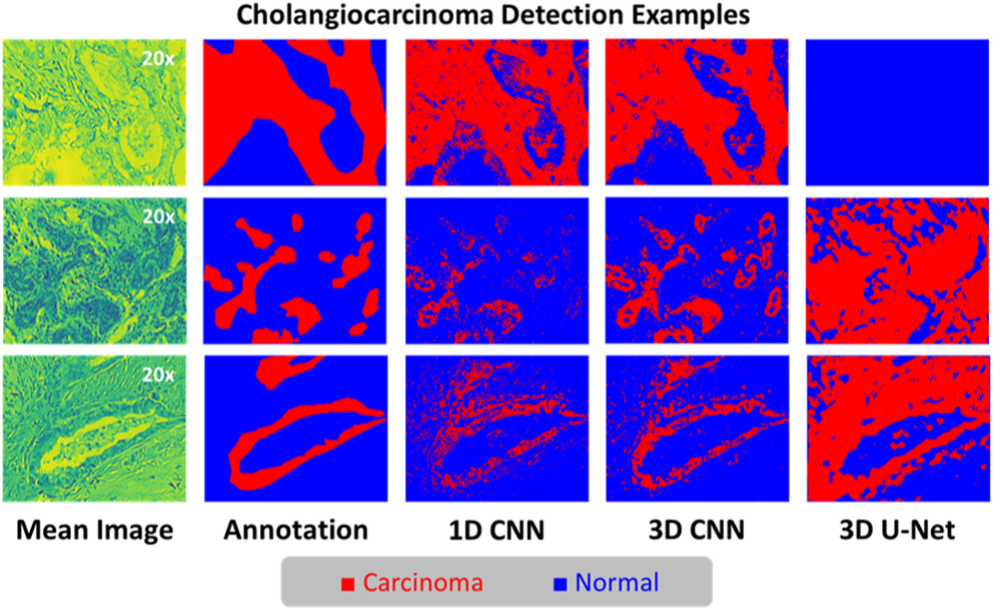
Example validation results of cholangiocarcinoma detection. There are 3 validation examples shown above, and each is with the mean spectral image, annotation, as well as the final segmentation outputs using 1D CCN, 3D CNN and 3D U-Net, respectively. In the cholangiocarcinoma detection task, there are 2 classes including carcinoma (red) and normal (blue).

**Figure 7:**
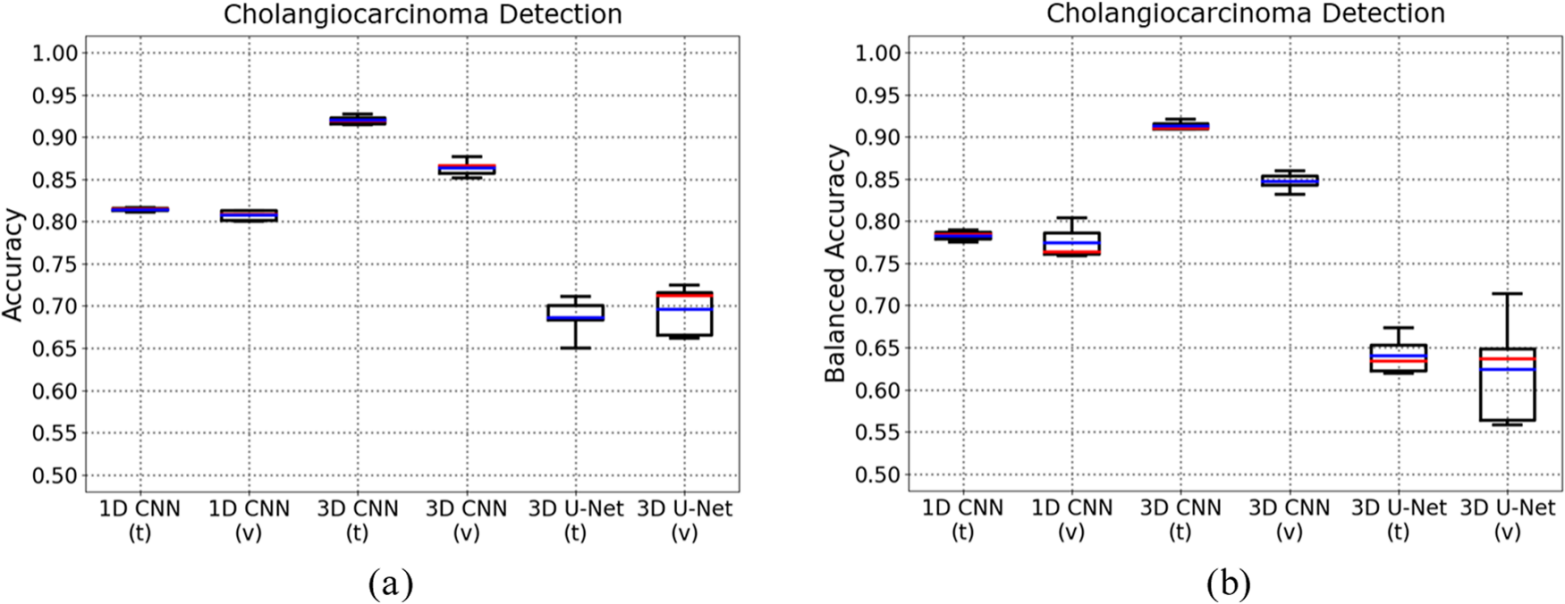
Network performance boxplots of cholangiocarcinoma detection. The boxplots above illustrate the training and validation performance of 1D CNN, 3D CNN, and 3D U-Net for cholangiocarcinoma detection using accuracy (7a) and balanced accuracy (7b), respectively. In the plots, “t” refers to “training”, and “v” refers to “validation”. These values were calculated for each fold, therefore each box contains 5 values. Besides, the red lines demonstrate the average values, while the blue lines demonstrate the median values.

In addition to the results on colorectal tissue data set reported above, some extra experiments were conducted on a cholangiocarcinoma data set as well for generalizability verification. Figures 6, 7 show the examples of segmentation outputs, and network performance boxplots, respectively. It is apparent that compared with 1D CNN, the 3D CNN preserved more details and avoided more artifacts, while the 3D U-Net had the worst performance. With only 200 hyperspectral scans, the average validation balanced accuracies of 1D CNN, 3D CNN, and 3D U-Net are 0.7745, 0.8470, and 0.6241, respectively. The proposed 3D CNN obtained validation balanced accuracies mostly around of 0.85, and those of 1D CNN fluctuated between 0.75 and 0.80, but the 3D U-Net just had unstable validation balanced accuracies from about 0.55 to 0.72. Furthermore, to compare network performance with the study of Yun et al.,^40^ Dice-Sørensen coefficient (DSC)^47^ is calculated for each model using the following eq 3
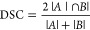
3where |*A*| represents the total number of positives predicted by the classifier, |*B*| represents the total number of actual positives in the annotation, and |*A*∩*B*| represents the number of true positives. Table 2 shows the DSC values of 1D CNN (57.76), 3D CNN (73.85), 3D U-Net (36.99) in this study, and some other competitors. Even though there were only 200 scans, the 3D CNN in this study still outperformed the RegNetX40 (70.88) and Swin-Transformer (72.10), and had comparable performance as the ResNet34 (75.44) proposed by Yun et al., who just used hard data set splitting without any cross-validation strategy.

**Table 2:** Comparison of Dice-Sørensen Coefficients for Cholangiocarcinoma Detection

	this study			Yun et al.		
	1D CNN	**3D CNN**	3D U-Net	ResNet34	RegNetX40	Swin-Transformer
DSC	57.76	**73.85**	36.99	75.44	70.88	72.10

After the experiments of epithelial tissue detection, colorectal cancer detection, as well as an extra cholangiocarcinoma detection for generalizability verification, it has been found that the 3D U-Net always lagged behind the 1D and 3D CNNs. To further investigate the reason for this issue, data amount and trainable parameters were also compared for each model. The results are shown in Table 3. For the epithelial tissue detection task, the 3D U-Net had an extremely low ratio of 3.8 × 10^–6^:1 between input data and trainable parameters, while the 1D and 3D CNNs had 15.9:1 and 1.7:1, respectively. Similarly, for colorectal cancer detection, this ratio of 3D U-Net was 2.8 × 10^–6^:1; and for the additional cholangiocarcinoma detection task, this ratio was still as low as 3.5 × 10^–3^:1 even after reducing the number of kernels to a half. It is generally recognized that deep learning algorithms are based on multilayered artificial neural networks, which require massive amounts of data to achieve high performance.^48^ Therefore, the data scarcity issue limits the practical performance of deep learning networks, even if some of them might have high potentials. In this way, although it is a common method in computer vision, the 3D U-Net could not perform satisfactorily due to the lack of training data, especially for biomedical applications.

**Table 3:** Comparison of Data Amount and Trainable Parameters

		1D CNN (inputs: 1D spectra)	3D CNN (inputs: 3D patches)	3D U-Net (inputs: 3D scans)
epithelial tissue detection	data count	1,018,640	1,018,640	749
	trainable parameters	63,956	569,812	2,597,636
	**ratio**	**15.9:1**	**1.7:1**	**3.8 × 10^–6^:1**
colorectal cancer detection	data amount	1,018,640	1,018,640	749
	trainable parameters	64,373	573,557	2,651,397
	**ratio**	**15.8:1**	**1.8:1**	**2.8 × 10^–6^:1**
cholangiocarcinoma detection	data amount	16,384,000	16,384,000	200
	trainable parameters	61,106	544,178	564,098
	**ratio**	**268.1:1**	**30.1:1**	**3.5 × 10^–3^:1**

## Conclusions

Because of the rapid rise of AI currently, deep learning algorithms play a more and more important role in various fields of research. Recently, these algorithms have already widely spread into data analysis models for Raman spectroscopy. However, most of the present methods only use 1D spectral data classification, instead of considering any neighboring information in space. In spite of some successes using this type of methods, ignoring spatially neighboring information is a huge loss of the 3D structure of Raman hyperspectral scans. Therefore, to investigate the feasibility of containing the spatial information on Raman spectroscopy for data analysis, spatially aware deep learning algorithms were implemented on a colorectal tissue data set with 3D Raman hyperspectral scans. This data set contains Raman spectra from normal, hyperplasia, adenoma, carcinoma tissues as well as artifacts. By rearranging the classes, classification tasks of 2 stages were performed on this data set: one is epithelial tissue detection, and the other is colorectal cancer detection. In both tasks, first, a simplified version of 3D U-Net was utilized for segmentation; second, another CNN network using 3D Raman patches was utilized for pixel-wise classification; third, the traditional 1D CNN method worked as baseline for comparison. Besides, to generate multiple results for performance analysis in a more statistical way, an external 5-fold cross-validation was applied for each method.

In terms of epithelial tissue detection, average values of validation balanced accuracies of 1D CNN, 3D CNN, and 3D U-Net are 0.946, 0.963, and 0.713, respectively; and these values for colorectal cancer detection are 0.926, 0.966, and 0.819, respectively. The proposed 3D CNN method had better and more stable performance than the conventional 1D CNN method. And all 3 deep learning networks outperformed the SVM-based method used by Vogler and co-workers. However, due to common data scarcity issue in biomedical research, the 3D U-Net method could not perform as desired with massive trainable parameters. On this colorectal tissue data set, the results of both tasks have confirmed that using spatially neighboring information on 3D Raman hyperspectral scans can increase the performance of deep learning models, although it might also increase some complexity of network training.

Apart from the colorectal tissue data set, experiments were also conducted on a cholangiocarcinoma data set for generalizability verification. The average validation balanced accuracies of 1D CNN, 3D CNN, and 3D U-Net are 0.774, 0.847, and 0.624, respectively. Besides, by calculating Dice-Sørensen coefficients, it is shown that the proposed 3D CNN method is comparable to the ResNet34 in the study of Yun et al., and it has outperformed their RegNetX40 and Swin-Transformer. Therefore, the findings in this study can also be potentially applied into other medical data sets with hyper-spectral scans, which greatly reduce the workload of further related studies, especially for spectroscopic data analysis in a spatially aware way.

In addition, the proposed method offers clinical advantages as well. Typically, the presence of a single cancer cell is enough for a patient to be diagnosed with cancer. Similarly, if just one pixel is classified as cancerous, it could result in a high rate of false positives, leading to overdiagnosis, which might cause problems including unnecessary treatments, increased anxiety, and higher medical costs, etc. Owing to the lack of spatial information, traditional 1D CNNs normally fail in this scenario with single spectra. But in this context, 3D U-Nets are usually less feasible due to their complex network architecture and limited size of medical data sets, despite their success in many other general tasks. Fortunately, the 3D CNN method proposed in this study is capable of resolving this issue by also checking spatial neighbors. Therefore, it is recommended to implement this method clinically for considering the spatial information in hyperspectral scans, which is usually collected but rarely used in analysis. This approach not only uses resources more effectively, but also helps prevent waste and improves clinical decision-making.

## Supporting Information Available

Additional example results and training process plots (PDF)

## References

[ref1] Raman C. V.; Krishnan K. S. A new type of secondary radiation. Nature 1928, 121, 501–502. 10.1038/121501c0.

[ref2] Ryabchykov O.; Guo S.; Bocklitz T. Photonic data analysis in 2050. Vib. Spectrosc. 2024, 132, 103685 10.1016/j.vibspec.2024.103685.

[ref3] Orlando A.; Franceschini F.; Muscas C.; et al. A comprehensive review on Raman spectroscopy applications. Chemosensors 2021, 9, 262 10.3390/chemosensors9090262.

[ref4] Li Z.; Deng L.; Kinloch I. A.; Young R. J. Raman spectroscopy of carbon materials and their composites: graphene, nanotubes and fibres. Prog. Mater. Sci. 2023, 135, 101089 10.1016/j.pmatsci.2023.101089.

[ref5] Cano-Trujillo C.; García-Ruiz C.; Ortega-Ojeda F. E.; et al. Forensic analysis of biological fluid stains on substrates by spectroscopic approaches and chemometrics: a review. Anal. Chim. Acta 2023, 1282, 341841 10.1016/j.aca.2023.341841. 37923402

[ref6] Lei L.; Massonnet G. Forensic analysis of white automotive paint of same manufacturer with Raman spectroscopy and chemometrics. J. Raman Spectrosc. 2024, 55 (2), 148–160. 10.1002/jrs.6626.

[ref7] Silge A.; Weber K.; Cialla-May D.; et al. Trends in pharmaceutical analysis and quality control by modern Raman spectroscopic techniques. TrAC, Trends Anal. Chem. 2022, 153, 116623 10.1016/j.trac.2022.116623.

[ref8] Yilmaz H.; Yilmaz D.; Taskin I. C.; Culha M. Pharmaceutical applications of a nanospectroscopic technique: surface-enhanced Raman spectroscopy. Adv. Drug Delivery Rev. 2022, 184, 114184 10.1016/j.addr.2022.114184. 35306126

[ref9] Xu Y.; Pan X.; Li H.; et al. Accuracy of Raman spectroscopy in the diagnosis of Alzheimer’s disease. Front. Psychiatry 2023, 14, 1112615 10.3389/fpsyt.2023.1112615. 37009107 PMC10060832

[ref10] Thomas K. M.; Ajithaprasad S.; Mithun N.; Pavithran M.S.; et al. Raman spectroscopy assisted tear analysis: a label free, optical approach for noninvasive disease diagnostics. Exp. Eye Res. 2024, 243, 109913 10.1016/j.exer.2024.109913. 38679225

[ref11] Guo S.; Popp J.; Bocklitz T. Chemometric analysis in Raman spectroscopy from experimental design to machine learning-based modeling. Nat. Protoc. 2021, 16 (12), 5426–5459. 10.1038/s41596-021-00620-3. 34741152

[ref12] Luo R.; Popp J.; Bocklitz T. Deep learning for Raman spectroscopy: a review. Analytica 2022, 3 (3), 287–301. 10.3390/analytica3030020.

[ref13] Qi Y.; Hu D.; Jiang Y.; et al. Recent progresses in machine learning assisted Raman spectroscopy. Adv. Opt. Mater. 2023, 11 (14), 2203104 10.1002/adom.202203104.

[ref14] Guo S.; Rösch P.; Popp J.; Bocklitz T. Modified PCA and PLS: towards a better classification in Raman spectroscopy-based biological applications. J. Chemom. 2020, 34 (4), e3202 10.1002/cem.3202.

[ref15] Othman N. H.; Radzol A. R. M.; Lee K. Y.; Mansor W. In Reduced Featured k-NN Classifier Model Optimal for Classification of Dengue Fever from Salivary Raman Spectra, Proceedings of 41st Annual International Conference of the IEEE Engineering in Medicine and Biology Society (EMBC), 2019; pp 471–474 10.1109/EMBC.2019.8856427. 31945940

[ref16] Chen X.; Wu X.; Chen C.; et al. Raman spectroscopy combined with a support vector machine algorithm as a diagnostic technique for primary Sjögren’s syndrome. Sci. Rep. 2023, 13, 5137 10.1038/s41598-023-29943-9. 36991016 PMC10060214

[ref17] Zhang W.; Giang C. M.; Cai Q.; et al. Using random forest for brain tissue identification by Raman spectroscopy. Mach. Learn.: Sci. Technol. 2023, 4 (4), 045053 10.1088/2632-2153/ad1349.

[ref18] Neo E. R. K.; Low J. S. C.; Goodship V.; Debattista K. Deep learning for chemometric analysis of plastic spectral data from infrared and Raman databases. Resour., Conserv. Recycl. 2023, 188, 106718 10.1016/j.resconrec.2022.106718.

[ref19] Tseng Y. M.; Chen K. L.; Chao P. H.; Han Y. Y.; Huang N. T. Deep learning-assisted surface-enhanced Raman scattering for rapid bacterial identification. ACS Appl. Mater. Interfaces 2023, 15 (22), 26398–26406. 10.1021/acsami.3c03212. 37216401

[ref20] Wang W.; Wang X.; Huang Y.; et al. Raman spectrum combined with deep learning for precise recognition of Carbapenem-resistant Enterobacteriaceae. Anal. Bioanal. Chem. 2024, 416 (10), 2465–2478. 10.1007/s00216-024-05209-9. 38383664

[ref21] He J.; Zhou R.; Ren P.; Li Y.; Xiong S. RepDwNet: lightweight deep learning model for special biological blood Raman spectra analysis. Chemosensors 2024, 12 (2), 29 10.3390/chemosensors12020029.

[ref22] Jensen E. A.; Serhatlioglu M.; Uyanik C.; et al. Label-free blood typing by Raman spectroscopy and artificial intelligence. Adv. Mater. Technol. 2024, 9, 2301462 10.1002/admt.202301462.

[ref23] He C.; Zhu S.; Wu S.; et al. Accurate tumor subtype detection with Raman spectroscopy via variational autoencoder and machine learning. ACS Omega 2022, 7, 10458–10468. 10.1021/acsomega.1c07263. 35382336 PMC8973095

[ref24] Shang L.; Tang J.; Wu J.; et al. Polarized micro-Raman spectroscopy and 2D convolutional neural network applied to structural analysis and discrimination of breast cancer. Biosensors 2023, 13, 65 10.3390/bios13010065. PMC9856190 36671896

[ref25] Zhu J.; Jiang X.; Rong Y.; et al. Label-free detection of trace level zearalenone in corn oil by surface-enhanced Raman spectroscopy (SERS) coupled with deep learning models. Food Chem. 2023, 414, 135705 10.1016/j.foodchem.2023.135705. 36808025

[ref26] Liu Y.; Gao Y.; Niu R.; et al. Rapid and accurate bacteria identification through deep-learning-based two-dimensional Raman spectroscopy. Anal. Chim. Acta 2024, 1332, 343376 10.1016/j.aca.2024.343376. 39580159

[ref27] Gonzalez R.; Woods R. Digital Image Processing, 4th ed.; Pearson International, 2017.

[ref28] Imani M.; Ghassemian H. An overview on spectral and spatial information fusion for hyperspectral image classification: current trends and challenges. Inf. Fusion 2020, 59, 59–83. 10.1016/j.inffus.2020.01.007.

[ref29] Datta D.; Mallick P. K.; Bhoi A. K.; et al. Hyperspectral image classification: potentials, challenges, and future directions. Comput. Intell. Neurosci. 2022, 2022 (3854635), 1–35. 10.1155/2022/3854635. PMC9071975 35528334

[ref30] Tejasree G.; Agilandeeswari L. An extensive review of hyperspectral image classification and prediction: techniques and challenges. Multimedia Tools Appl. 2024, 83, 80941–81038. 10.1007/s11042-024-18562-9.

[ref31] Gao W.; Liu F.; Liu J.; Xiao L.; Tang X. Spatial-spectral adaptive learning with pixelwise filtering for hyperspectral image classification. IEEE Trans. Geosci. Electron. 2023, 61, 1–15. 10.1109/TGRS.2023.3284074.

[ref32] Scheibenreif L.; Mommert M.; Borth D. Masked Vision Transformers for Hyperspectral Image Classification, Proceedings of 2023 IEEE/CVF Conference on Computer Vision and Pattern Recognition Workshops (CVPRW), 2023; pp 2166–2176 10.1109/CVPRW59228.2023.00210.

[ref33] Ashraf M.; Alharthi R.; Chen L.; et al. Attention 3D central difference convolutional dense network for hyperspectral image classification. PLoS One 2024, 19 (4), e0300013 10.1371/journal.pone.0300013. 38598444 PMC11006129

[ref34] Singh S. P.; Wang L.; Gupta S.; et al. 3D deep learning on medical images: a review. Sensors 2020, 20 (18), 5097 10.3390/s20185097. 32906819 PMC7570704

[ref35] Fernando K. R. M.; Tsokos C. P. Deep and statistical learning in biomedical imaging: state of the art in 3D MRI brain tumor segmentation. Inf. Fusion 2023, 92, 450–465. 10.1016/j.inffus.2022.12.013.

[ref36] Di Napoli A.; Tagliente E.; Pasquini L.; et al. 3D CT-inclusive deep-learning model to predict mortality, ICU admittance, and intubation in COVID-19 patients. J. Digital Imaging 2023, 36, 603–616. 10.1007/s10278-022-00734-4. PMC9713092 36450922

[ref37] Vogler N.; Bocklitz T.; Salah F. S.; et al. Systematic evaluation of the biological variance within the Raman based colorectal tissue diagnostics. J. Biophotonics 2016, 9 (5), 533–541. 10.1002/jbio.201500237. 26687775

[ref38] Çiçek Ö.; Abdulkadir A.; Lienkamp S. S.; Brox T.; Ronneberger O. 3D U-Net: Learning Dense Volumetric Segmentation from Sparse AnnotationProceedings of 19th International Conference on Medical Image Computing and Computer-Assisted Intervention (MICCAI)2016; pp 424–432 10.1007/978-3-319-46723-8_49.

[ref39] Zhang Q.; Li Q.; Yu G.; et al. A multidimensional choledoch database and benchmarks for cholangiocarcinoma diagnosis. IEEE Access 2019, 7, 149414–149421. 10.1109/ACCESS.2019.2947470.

[ref40] Yun B.; Li Q.; Mitrofanova L.; Zhou C.; Wang Y. Factor space and spectrum for medical hyperspectral image segmentation. In Medical Image Computing and Computer-Assisted Intervention (MICCAI), Lecture Notes in Computer Science, 2023; Vol. 14223, pp 152–162 10.1007/978-3-031-43901-8_15.

[ref41] Storozhuk D.; Ryabchykov O.; Popp J.; Bocklitz T. RAMANMETRIX: a delightful way to analyze Raman spectra arXiv 2022, pp 1–30 10.48550/arXiv.2201.07586.

[ref42] Ryan C. G.; Clayton E.; Griffin W. L.; et al. SNIP, a statistics-sensitive background treatment for the quantitative analysis of PIXE spectra in geoscience applications. NIM-B 1988, 34 (3), 396–402. 10.1016/0168-583X(88)90063-8.

[ref43] Luo R.; Guo S.; Bocklitz T. In Sample Size Estimation of Transfer Learning for Colorectal Cancer Detection, Proceedings of 13th International Conference on Pattern Recognition Applications and Methods (ICPRAM), 2024; pp 841–851 10.5220/0012449500003654.

[ref44] Kingma D. P.; Ba J. L. In Adam: A Method for Stochastic Optimization, Proceedings of 3rd International Conference on Learning Representations (ICLR), 2015; pp 1–15 10.48550/arXiv.1412.6980.

[ref45] Guo S.; Bocklitz T.; Neugebauer U.; Popp J. Common mistakes in cross-validating classification models. Anal. Methods 2017, 9 (30), 4410–4417. 10.1039/C7AY01363A.

[ref46] Luo R.; Bocklitz T. A systematic study of transfer learning for colorectal cancer detection. Inf. Med. Unlocked 2023, 40, 101292 10.1016/j.imu.2023.101292.

[ref47] Carass A.; Roy S.; Gherman A.; et al. Evaluating white matter lesion segmentations with refined Sørensen-Dice analysis. Sci. Rep. 2020, 10, 8242 10.1038/s41598-020-64803-w. 32427874 PMC7237671

[ref48] Goodfellow I.; Bengio Y.; Courville A. Deep Learning; MIT Press, 2016.

