# Supplementary material for: 3D Hyperspectral Data Analysis with Spatially Aware Deep Learning for Diagnostic Applications
Source: Anal Chem. 2025 Apr 3;97(14):7729–37. doi: 10.1021/acs.analchem.4c05549 (PMC12004353; doi:10.1021/acs.analchem.4c05549)

Supporting Information

3D Hyperspectral Data Analysis with Spatially Aware Deep Learning for Diagnostic Applications

Ruihao Luo[1,2], Shuxia Guo[1,2], Julian Hniopek[1,2], and Thomas Bocklitz[1,2,*]

1. Institute of Physical Chemistry (IPC) and Abbe School of Photonics (ASP), Friedrich-Schiller-Universität Jena, Helmholtzweg 4, 07743 Jena, Germany
2. Leibniz Institute of Photonic Technology (IPHT), Albert-Einstein-Straße 9, 07745 Jena, Germany

* Corresponding E-mail: thomas.bocklitz@uni-jena.de

Table of Contents

Supplementary Tables

Table S1. Additional Examples of Epithelial Tissue Detection S2

Table S2. Additional Examples of Colorectal Cancer Detection S3

Table S3. Additional Examples of Cholangiocarcinoma Detection S4

Supplementary Figure

Figure S1. Training Process Plots S5

Table S1. Additional Examples of Epithelial Tissue Detection

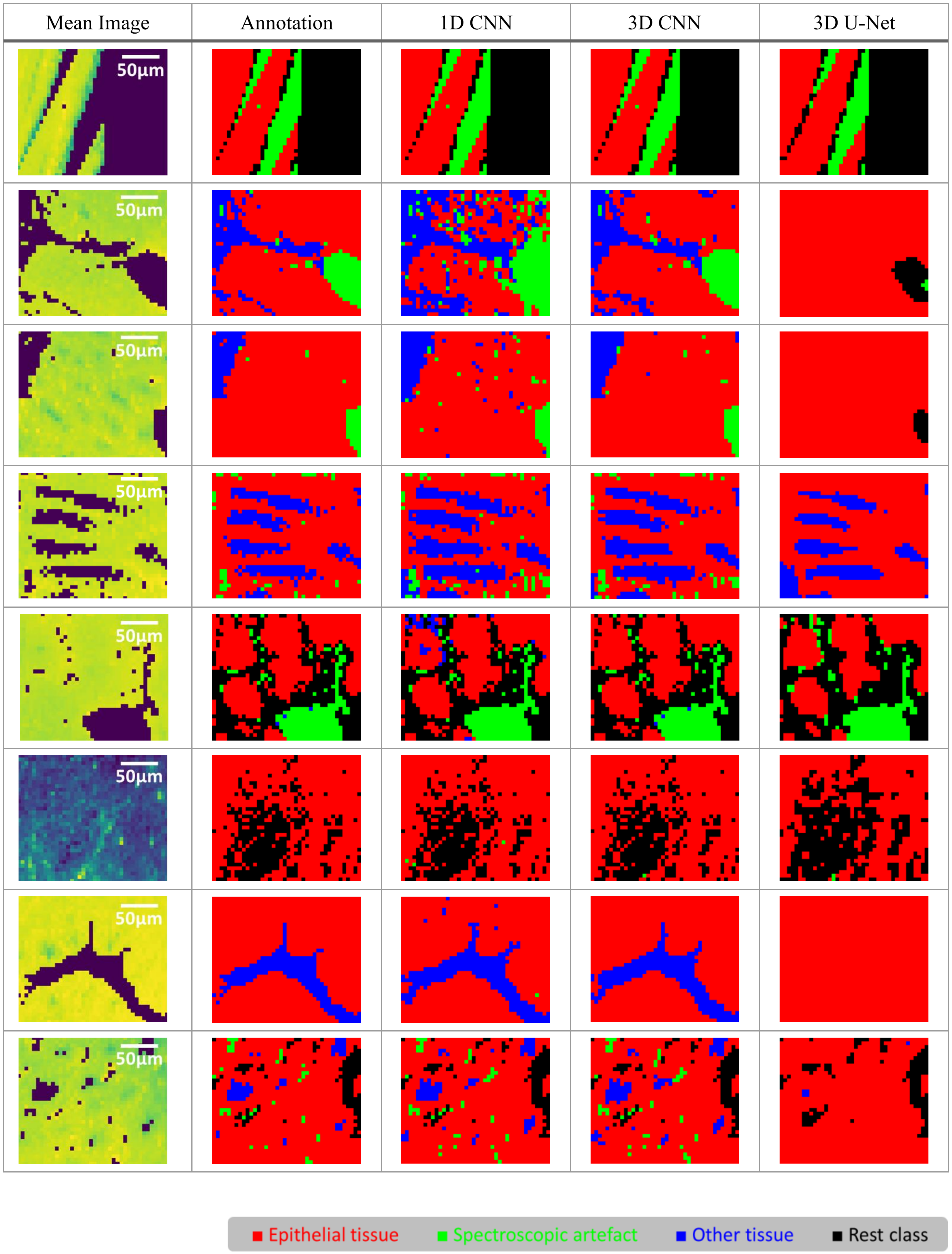

Table S2. Additional Examples of Colorectal Cancer Detection

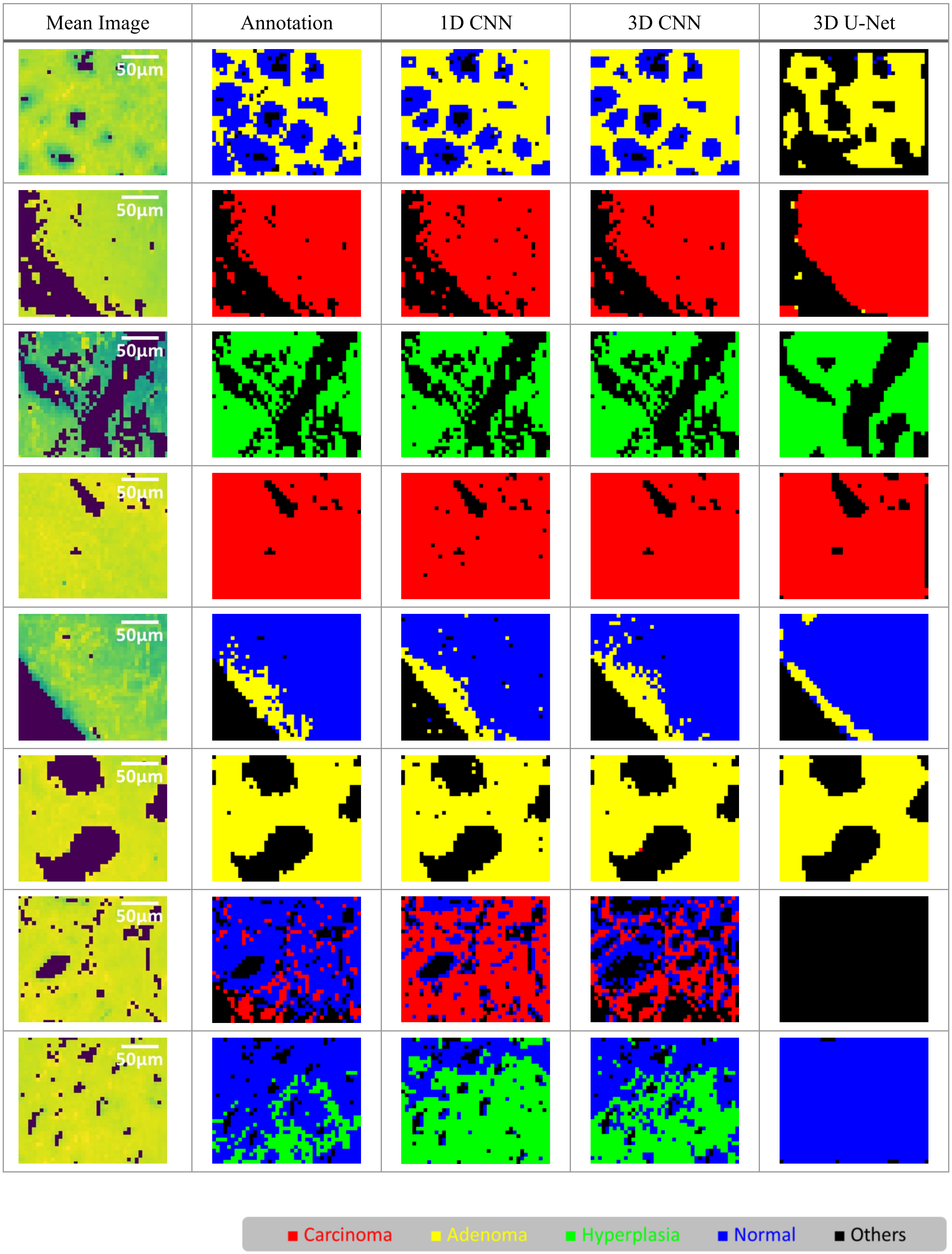

Table S3. Additional Examples of Cholangiocarcinoma Detection

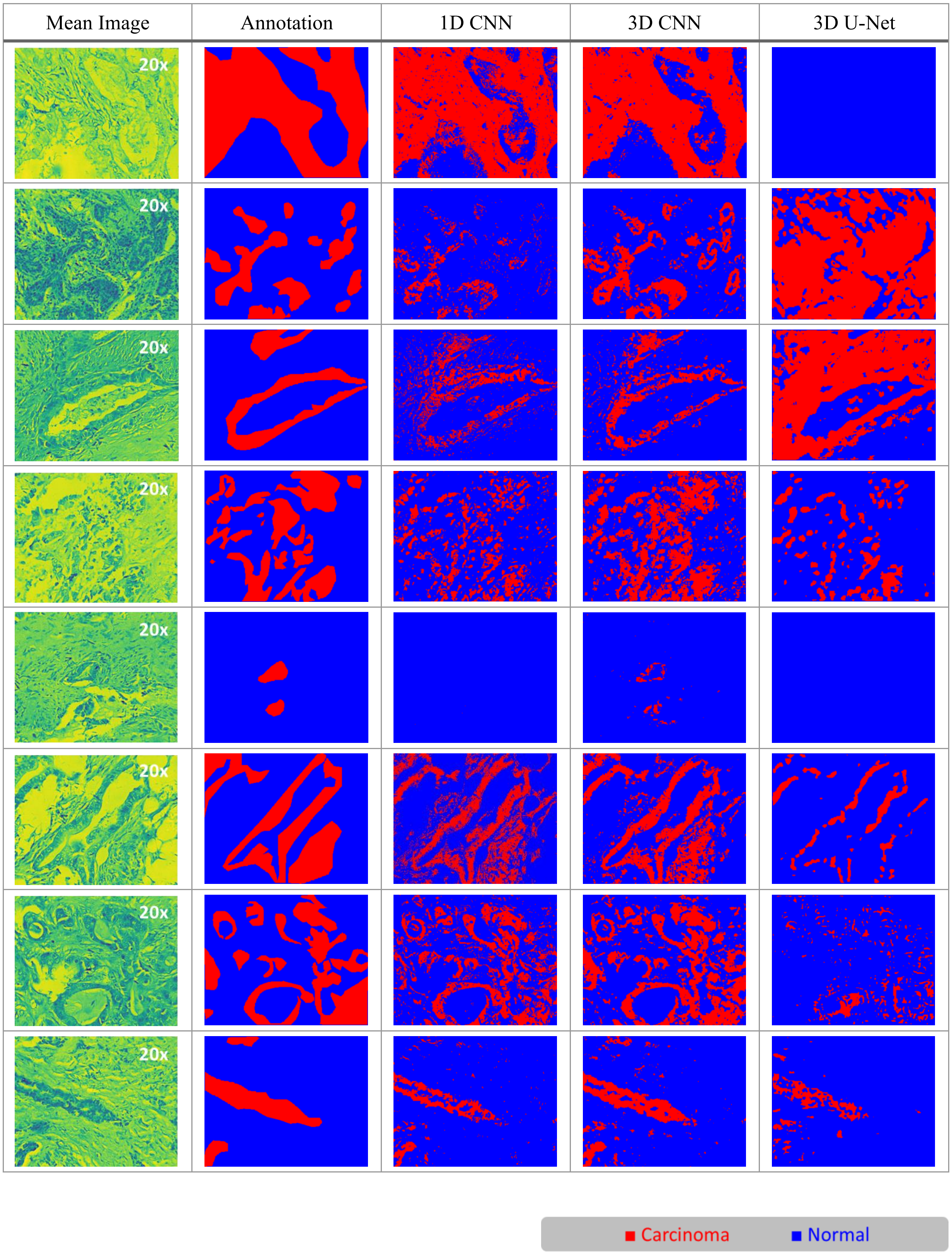

Figure S1. Training Process Plots

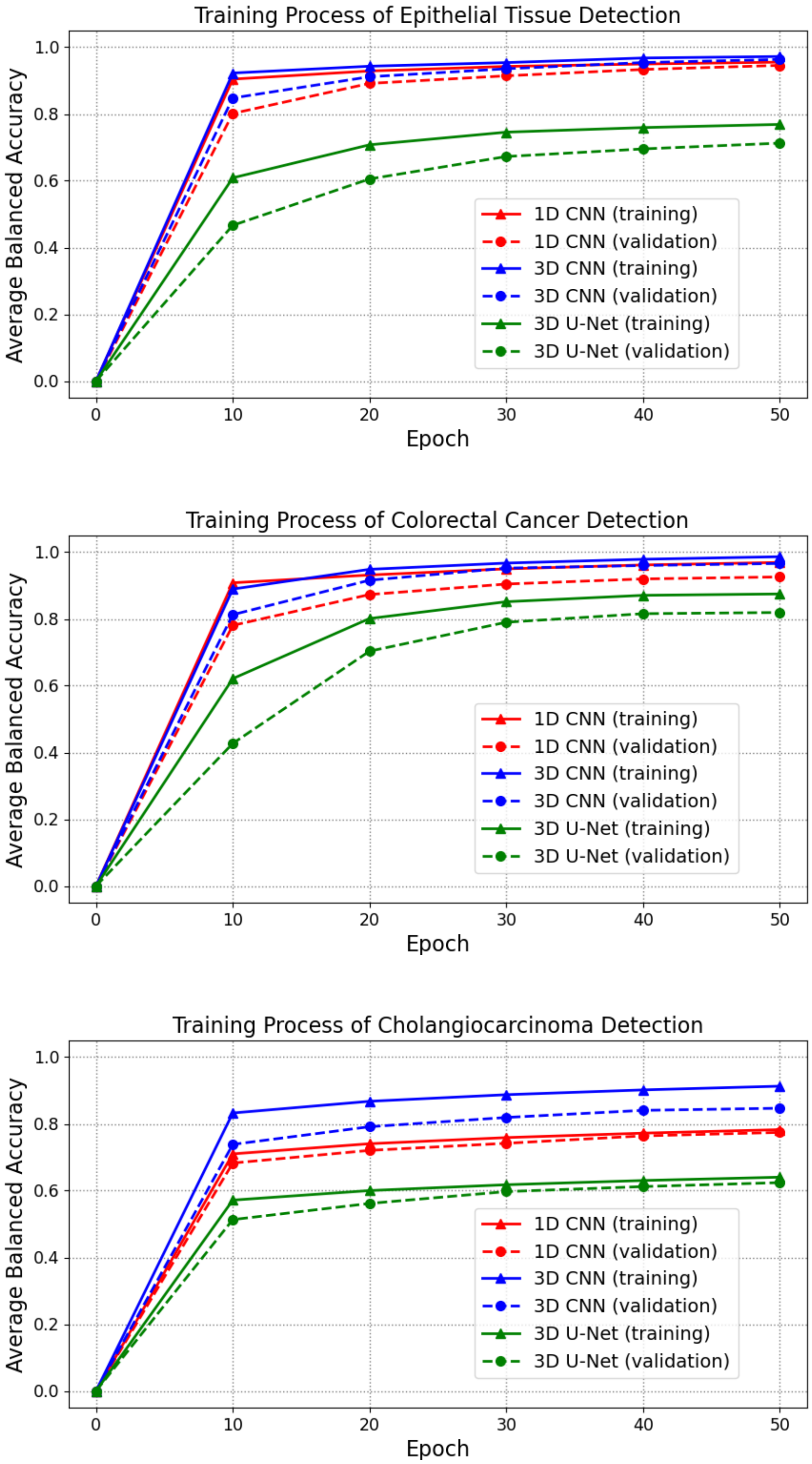

Supplement: Supplementary file 1 — ac4c05549_si_001.pdf [file ac4c05549_si_001.pdf]
